# ﻿Two new species of the dwarf centipede genus *Nannarrup* Foddai, Bonato, Pereira & Minelli, 2003 (Chilopoda, Geophilomorpha, Mecistocephalidae) from Japan

**DOI:** 10.3897/zookeys.1115.83946

**Published:** 2022-08-01

**Authors:** Sho Tsukamoto, Satoshi Shimano, Katsuyuki Eguchi

**Affiliations:** 1 Systematic Zoology Laboratory, Graduate School of Science, Tokyo Metropolitan University, Minami-osawa 1-1 Hachioji-shi, Tokyo 192-0397, Japan Tokyo Metropolitan University Tokyo Japan; 2 Science Research Center, Hosei University, Fujimi 2-17-1 Chiyoda-ku, Tokyo 102-8160, Japan Hosei University Tokyo Japan; 3 Department of International Health and Medical Anthropology, Institute of Tropical Medicine, Nagasaki University, Sakamoto 1-12-4, Nagasaki, Nagasaki 852-8523, Japan Nagasaki University Nagasaki Japan

**Keywords:** Description, DNA barcoding, molecular phylogeny, morphology, sex ratio, taxonomy

## Abstract

The genus *Nannarrup* Foddai, Bonato, Pereira & Minelli, 2003 is a monotypic genus established on the basis of the possibly introduced species *N.hoffmani* Foddai, Bonato, Pereira & Minelli, 2003, from New York, USA. In the present study, in a field survey conducted throughout Japan, *Nannarrup*-like specimens were collected from Honshu, Shikoku, and Kyushu. These specimens clearly showed the diagnostic characteristics of the genus but were morphologically distinct from *N.hoffmani*. Furthermore, morphological analysis and DNA barcoding revealed that these specimens could be assigned to two distinct undescribed species. On the basis of these results, *N.innuptus* Tsukamoto, **sp. nov.** and *N.oyamensis* Tsukamoto, **sp. nov.** are described. The three *Nannarrup* species can be distinguished from each other on the basis of the following combination of characteristics: presence or absence of a pair of smooth or weakly areolate areas along the posterior part of the paraclypeal sutures; the width-to-length ratio of the denticle on the trochanteroprefemur; the pigmentation of the denticle on the tarsungulum. Moreover, the field survey resulted in the collection of exclusively female specimens of *N.innuptus* Tsukamoto, **sp. nov.**, which shows the possibility of parthenogenesis of this species.

## ﻿Introduction

The geophilomorph family Mecistocephalidae Bollman, 1893, is a distinct monophyletic group, which is well characterized morphologically by a cephalic capsule and the forcipular segment that are obviously sclerotized and darker than the remaining trunk segments, the mandible with a series of pectinate lamellae only, trunk sternites with an internal apodeme, a mid-longitudinal sulcus, and the intraspecific invariance in the segment number, except in some species of the genus *Mecistocephalus* Newport, 1843 (Bonato et al. 2003, 2014; Uliana et al. 2007; Bonato 2011). The family is distributed mainly in tropical and subtropical regions and especially diversified at the species and higher phylogenetic levels in East Asia (Uliana et al. 2007; Bonato 2011). The family Mecistocephalidae comprises three subfamilies: Mecistocephalinae Bollman, 1893; Dicellophilinae Cook, 1896; and Arrupinae Chamberlin, 1912.

The subfamily Arrupinae comprises four valid genera: *Arrup* Chamberlin, 1912; *Agnostrup* Foddai, Bonato, Pereira & Minelli, 2003; *Partygarrupius* Verhoeff, 1939; *Nannarrup* Foddai, Bonato, Pereira & Minelli, 2003. Arrupinae has been reported mainly in East Asia and is diversified at the species level in Japan (Foddai et al. 2003; Uliana et al. 2007). To date, 21 species are reported in East Asia (18 spp.), Central Asia (1 sp.), California (1 sp.), New York (1 sp.), and 11 species are reported in Japan (Bonato et al. 2016).

The genus *Nannarrup* was established for a single species, *N.hoffmani* Foddai, Bonato, Pereira & Minelli, 2003. The genus has peculiar morphological characteristics (for details, see the section “Taxonomic account”), which likely evolved as a result of miniaturization. *Nannarruphoffmani* was originally described on the basis of specimens from New York City, USA. Foddai et al. (2003) stated that the species has been definitely introduced from western America or East Asia. However, the native range of the species and the genus remain unknown.

In field surveys in Honshu, Shikoku and Kyushu, Japan (2017–2022), the authors of the present study (ST and KE) collected 88 mecistocephalid specimens, which clearly showed the diagnostic characteristics of the genus *Nannarrup*. However, these “*Nannarrup*-like” specimens can be distinguished from *N.hoffmani* by the shape of the denticle on the forcipular trochanteroprefemur. Therefore, the present study aimed to assign the Japanese “*Nannarrup*-like” specimens to the current classification of Arrupinae using an integrative approach of morphological analysis and DNA barcoding, using the mitochondrial *COI* and *16S* ribosomal RNA genes, and the nuclear *28S* ribosomal RNA genes.

## ﻿Materials and methods

### ﻿Taxon sampling

Eighty-eight “*Nannarrup*-like” specimens, including 13 juveniles (in which sex determination is not possible), were collected by hand from Honshu (Aomori, Akita, Iwate, Yamagata, Fukushima, Niigata, Tokyo, Kanagawa, Shizuoka, Wakayama, Hyogo, Okayama, and Yamaguchi prefectures), Shikoku (Kochi and Ehime prefectures), and Kyushu (Fukuoka, Miyazaki, and Kagoshima prefectures). These specimens were included as ingroup in the present study. Each specimen was specified by its own specimen identification number in the form “TSYYYYMMDD-XX,” where TS is an abbreviation of the first author’s name, Tsukamoto Sho; YYYYMMDD designates the date on which the specimen was collected; XX is the identification number assigned to each specimen collected on a particular date (e.g., TS20171010-01).

**Figure 1.**
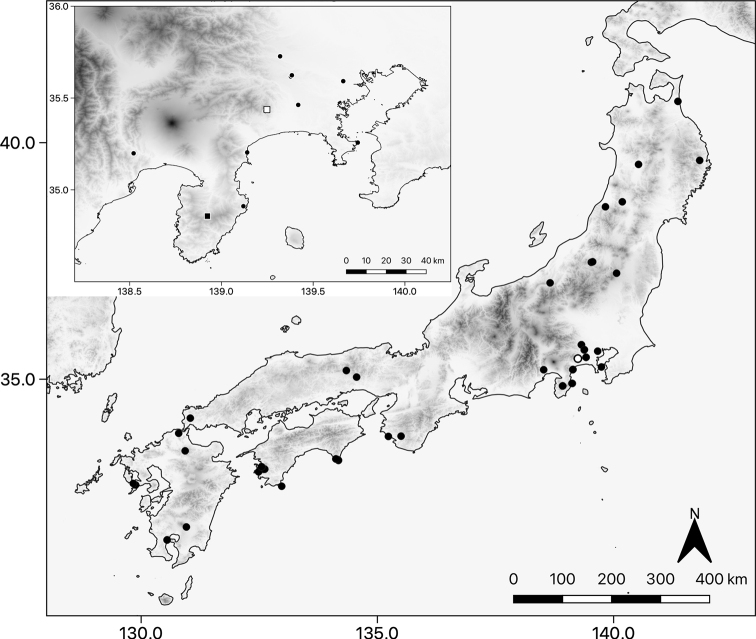
Map of collection sites of specimens examined in the present study. Black circle, *Nannarrupinnuptus* sp. nov.; black square, the type locality of *N.innuptus* sp. nov.; white circle, *N.oyamensis* sp. nov.; white square, the type locality of *N.oyamensis* sp. nov.

Type specimens of *Nannarrup* were deposited at the Collection of Myriapoda, Department of Zoology, National Museum of Nature and Science, Tokyo (**NSMT**), and Museum of Nature and Human Activities, Hyogo (**MNHAH**). See the “Taxonomic account” section for the deposition site of each type specimen. All non-type voucher specimens of *Nannarrup* are retained by the first author. The collection sites of examined specimens are shown in Fig. 1 and Taxonomic account section. The altitude data provided by AW3D of JAXA (https://www.eorc.jaxa.jp/ALOS/jp/index_j.htm) and the coastal line provided by the digital nation land information (https://nlftp.mlit.go.jp/index.html) were used for generating Fig. 1.

### ﻿Morphological analysis

Cephalic capsule, maxillae, mandibles, the forcipular segment and leg-bearing segments were made transparent using lactic acid or the Chelex-TE protocol to examine the anatomy and produce images (Satria et al. 2015). Cephalic capsule and maxillae of some specimens were mounted in Hoyer’s medium (gum arabic, chloral hydrate, and glycerol) or Euparal. Multi-focused images of these body parts were produced using Helicon Focus Pro 6.7.1 (https://www.heliconsoft.com/helicon-focus-history-of-changes-win/) from a series of source images taken using a Canon EOS Kiss X9 digital camera attached to a Nikon AZ100 microscope and improved using Adobe Photoshop Elements 10. Body parts were then measured directly using an ocular micrometer attached to the microscope or by measuring on the basis of the images using ImageJ software (http://imageJ.nih.gov/ij/). The morphological terminology used in the present study is mainly derived from Bonato et al. (2010). Specimens with fully developed paired gonopods—that is, evidently bi-articulated in males and touching each other in females—were determined to be adults, and those with incompletely developed paired gonopods were determined to be subadults; specimens without gonopods were determined to be juveniles.

### ﻿DNA sequencing

Genomic DNA was extracted from one or two legs, the head, or a body segment of each specimen by following the Chelex-TE-ProK protocol (Satria et al. 2015) with incubation for 24 h.

PCR amplification was performed in a MiniAmp Thermal Cycler (Thermo Fisher Scientific, Waltham, Massachusetts, USA) in a 10.5-µL reaction volume containing 5 µL 2× PCR buffer for KOD FX Neo, 2 µL of 2 mM dNTPs, 0.3 µL of 10 pmol/µL forward and reverse primers, 0.2 µL of 1.0 U/µL DNA polymerase KOD FX Neo (TOYOBO KFX-201X5), and 1.0 µL DNA template. The sequences of primers for the mitochondrial *COI* and *16S* and nuclear *28S* genes are shown in Table 1. Each PCR product was screened by electrophoresis on 2.0% agarose gel in 1× TAE.

**Table 1.** The list of primers used in the present study.

Genes	Primer name	Sequence (5' - 3')	Source
*COI*	LCO-CH	TTT CAA CAA AYC AYA AAG ACA TYG G	Tsukamoto et al. (2021)
	HCO-CH	TAA ACT TCT GGR TGR CCR AAR AAT CA	
*16S rRNA*	16Sa	CGC CTG TTT ATC AAA AAC AT	Xiong and Kocher (1991)
	16Sbi	CTC CGG TTT GAA CTC AGA TCA	
*28S rRNA*	28S D1F	GGG ACT ACC CCC TGA ATT TAA GCA T	Boyer and Giribet (2007)
	28S rD4b	CCT TGG TCC GTG TTT CAA GAC	Edgecombe and Giribet (2006)

Amplification conditions for mitochondrial *COI* were as follows: 98 °C for 2 min; 5 cycles of 98 °C for 10 s, 45 °C for 30 s, and 68 °C for 45 s; 40 cycles of 98 °C for 10 s, 48.5 °C for 30 s (annealing step), and 68 °C for 45 s; and 68 °C for 7 min. If the target fragment of *COI* was not appropriately amplified, the annealing temperature was changed from 48.5 °C to 50 °C, and PCR was performed again by omitting the first five cycles of annealing and the extension step.

Amplification conditions for mitochondrial *16S* were as follows: 98 °C for 2 min; 35 cycles of 98 °C for 10 s, 45 °C for 30 s (annealing step), and 68 °C for 45 s; and 68 °C for 7 min. If the target fragment of *16S* was not appropriately amplified, the annealing temperature was changed from 45 °C to 48 °C, and the number of annealing cycles was changed from 35 to 45.

Amplification conditions for nuclear *28S* were as follows: 98 °C for 2 min; 5 cycles of 98 °C for 10 s, 42 °C for 30 s, and 68 °C for 1 min; 30 cycles of 98 °C for 10 s, 50 °C for 30 s (annealing step), and 68 °C for 1 min; and 68 °C for 7 min. If the target fragment of *28S* was not appropriately amplified, the annealing temperature was changed from 50 °C to 48 °C, and the number of annealing cycles was changed from 30 to 40–45 cycles. Furthermore, PCR was performed again by omitting the first five cycles of annealing and the extension step.

The amplified products were incubated at 37 °C for 30 min and 80 °C for 20 min with IllustraTM ExoStar (GE Healthcare, Buckinghamshire, UK) to remove any excess primers and nucleotides. All nucleotide sequences were determined by direct sequencing using ABI PRISM BigDye Terminator Cycle Sequencing Kit ver. 3.1 (Thermo Fisher Scientific) or BrilliantDyeTM Terminator Cycle Sequencing Kit v. 3.1 (Nimagen, B.V., Nijmegen, Netherlands) with an ABI 3130xl automated sequencer (Thermo Fisher Scientific). The sequences were assembled using ChromasPro 1.7.6 (Technelysium Pty Ltd., Australia) and deposited in the databases DDBJ, EMBL, and GenBank under the accession numbers LC715482–LC715706 (Table 2).

**Table 2.** The list of specimens that were used in the phylogenetic analyses.

Species	Accession No.			Reference
	COI	16S	28S	
*Nannarrupinnuptus* sp. nov.	LC715482–LC715554	LC715557–LC715629	LC715632–LC715704	the present study
*Nannarrupoyamensis* sp. nov.	LC715455–LC715556	LC715530–LC715631	LC715605–LC715706	the present study
*Dicellophiluscarniolensis* (C.L. Koch, 1847)	KF569305	HM453225	HM453285	Murienne et al. (2010), Bonato et al. (2014)
*Mecistocephalusguildingii* Newport, 1843	AY288747	AY288728	HM453283	Edgecombe and Giribet (2004), Murienne et al. (2010)
*Mecistocephalussubgigas* (Silvestri, 1919)	AF370837	AF370862	HM453284	Giribet et al. (2001), Murienne et al. (2010)

### ﻿Molecular phylogenetic analyses

The sequences obtained using the methods described above were used for phylogenetic analyses; the *COI*, *16S*, and *28S* sequences of the mecistocephalid species *Dicellophiluscarniolensis* C.L. Koch, 1847; *Mecistocephalusguildingii* Newport, 1843; and *Mecistocephalussubgigas* (Silvestri, 1919), obtained from GenBank were used as outgroups (Table 2).

All sequences were aligned using MAFFT v. 7.475 (Katoh and Standley 2013). For *COI*, the alignment was performed using the default setting. For *16S* and *28S*, secondary structure alignment was performed using the X-INS-i option.

Maximum-likelihood (**ML**) trees were created on the basis of the sequence dataset for each gene using IQ-tree 1.6.12 (Nguyen et al. 2015). In the ML analysis for the *COI* dataset, TN + F was selected for the first codon position, TNe + G4 was selected for the second codon position, and F81 + F was selected for the third codon position as the optimal substitution model according to the Bayesian information criterion (BIC). In the ML analysis for the *16S* dataset, TIM3 + F + I + G4 was selected as the optimal substitution model according to BIC. In the ML analysis for the *28S* dataset, TIM3e + G4 was selected as the optimal substitution model according to BIC. Ultrafast bootstrap analysis (**UFBoot**; Hoang et al. 2018) and SH-like approximate likelihood ratio test (**SH-aLRT**; Guindon et al. 2010) were performed with 1,000 replicates.

### ﻿Calculation of genetic distances

Aligned datasets (*COI*, *16S*, and *28S*) used for phylogenetic analyses were also used to calculate the genetic distances. Pairwise p-distances and Kimura-two-parameter (K2P) distances were calculated for each of the three genes of “*Nannarrup*-like” specimens using MEGA X (Kumar et al. 2018) using the setting “pairwise deletion.”

## ﻿Results

### ﻿Taxonomic position of “*Nannarrup*-like” specimens

All 88 “*Nannarrup*-like” specimens collected in Japan possessed the diagnostic characteristics of the subfamily Arrupinae (Bonato et al. 2003): body tapering backwards; leg-bearing trunk uniform in color, without dark patches; clypeus with 11–17 clypeal setae, placed in two lateral areas; internal margin of labral anterior ala reduced to a pointed end; posterior alae without longitudinal stripes; posterior margin of labral side-piece sinuous (and with short fringe, in contrast to “diagnosis” of Arrupinae in Bonato et al (2003)); cerrus absent.

Furthermore, these “*Nannarrup*-like” specimens possessed the diagnostic characteristics of the genus *Nannarrup* established by Foddai et al. (2003) on the basis of *N.hoffmani*: body length ca 10-mm long in adults, with 41 pairs of legs; frontal line absent; clypeus with two small plagulae; clypeal ratio ca 1:6–1:7; cephalic pleurites with a pair of short stili, without setae and a pair of spicula; side pieces of labrum subdivided into anterior and posterior alae; mandible with four well-developed pectinate lamellae; first maxillae separated from each other by a longitudinal line at the coxosternite; second maxillae not separated from each other at the coxosternite, with metameric pores close to the posterior margin, without claw; forcipular telopodites not reaching the anterior margin of the head in the closed position; trochanteroprefemur with single distal denticle; forcipular femur without teeth; tarsungulum with basal, well-developed denticle; forcipular tergite without median sulcus; sternites with sulcus, which is not anteriorly furcate; last metasternite subtriangular; ventral surface of each coxopleurite with numerous pores; anal pores present.

These “*Nannarrup*-like” specimens (and even *N.hoffmani*) can be distinguished from the genus *Arrup*, which is speciose in the Japanese Archipelago (Uliana et al. 2007), by first maxillae separated from each other by a longitudinal line at the coxosternite. In addition, they can be distinguished from *Agnostrup* and *Partygarrupius*, that are also known from Japan, by two small clypeal plagulae (plagulae of *Agnostrup* and *Partygarrupius* covered one-half to most of their clypeus (Foddai et al. 2003; Uliana et al. 2007)). Therefore, these “*Nannarrup*-like” specimens were confidently assigned to *Nannarrup*.

### ﻿Morphological comparison between Japanese *Nannarrup* and *N.hoffmani*

The Japanese *Nannarrup* specimens were distinguishable from *N.hoffmani* on the basis of two morphological characteristics: the width-to-length ratio of the denticle of the trochanteroprefemur, which was 1:0.53 in *N.hoffmani* (measured from fig. 14 in Foddai et al. (2003)) but 1:1.3–1.6 in the Japanese specimens; pigmentation of the denticle on the tarsungulum was less prominent than that of the denticle on the trochanteroprefemur in *N.hoffmani* but equivalent to that in the Japanese specimens (Foddai et al. 2003).

In two specimens from Kanagawa prefecture, in the Kanto Region of Japan (TS20210217-04 and TS20210725-02), “two additional smooth areas along the posterior part of the paraclypeal sutures” in the clypeus (sensu Foddai et al. (2003)) were not observed. However, this characteristic was observed in the remaining Japanese *Nannarrup* specimens.

Therefore, the Japanese *Nannarrup* specimens were divided into two morphospecies, namely *N.* sp. 1 and *N.* sp. 2 (see Table 3), both of which were morphologically distinct from *N.hoffmani*.

**Table 3.** Morphological comparison among three species of the genus *Nannarrup*.

Species	Clypeus	Forcipule	
	Two additional smooth areas along paraclypeal sutures	The width to length ratio of the denticle on trochanteroprefemur	Pigmentation of the tooth on the tarsungulum
*Nannarrupinnuptus* sp. nov. (= *Nannarrup* sp. 1)	+	1: 1.3–1.6	equal to the denticle on trochanteroprefemur
*Nannarrupoyamensis* sp. nov. (= *Nannarrup* sp. 2)	-	1: 1.3	equal to the denticle on trochanteroprefemur
*Nannarruphoffmani* Foddai, Bonato, Pereira & Minelli, 2003	+	1: 0.5	slighter than the denticle on trochanteroprefemur

### ﻿Molecular phylogenetic analyses

The *COI*, *16S*, and *28S* sequences were successfully determined for 73 specimens of *Nannarrup* sp. 1 (except for two specimens from Tokyo, i.e., TS20171010-01 and TS20180627-01) and both specimens of *N.* sp. 2.

**Figure 2.**
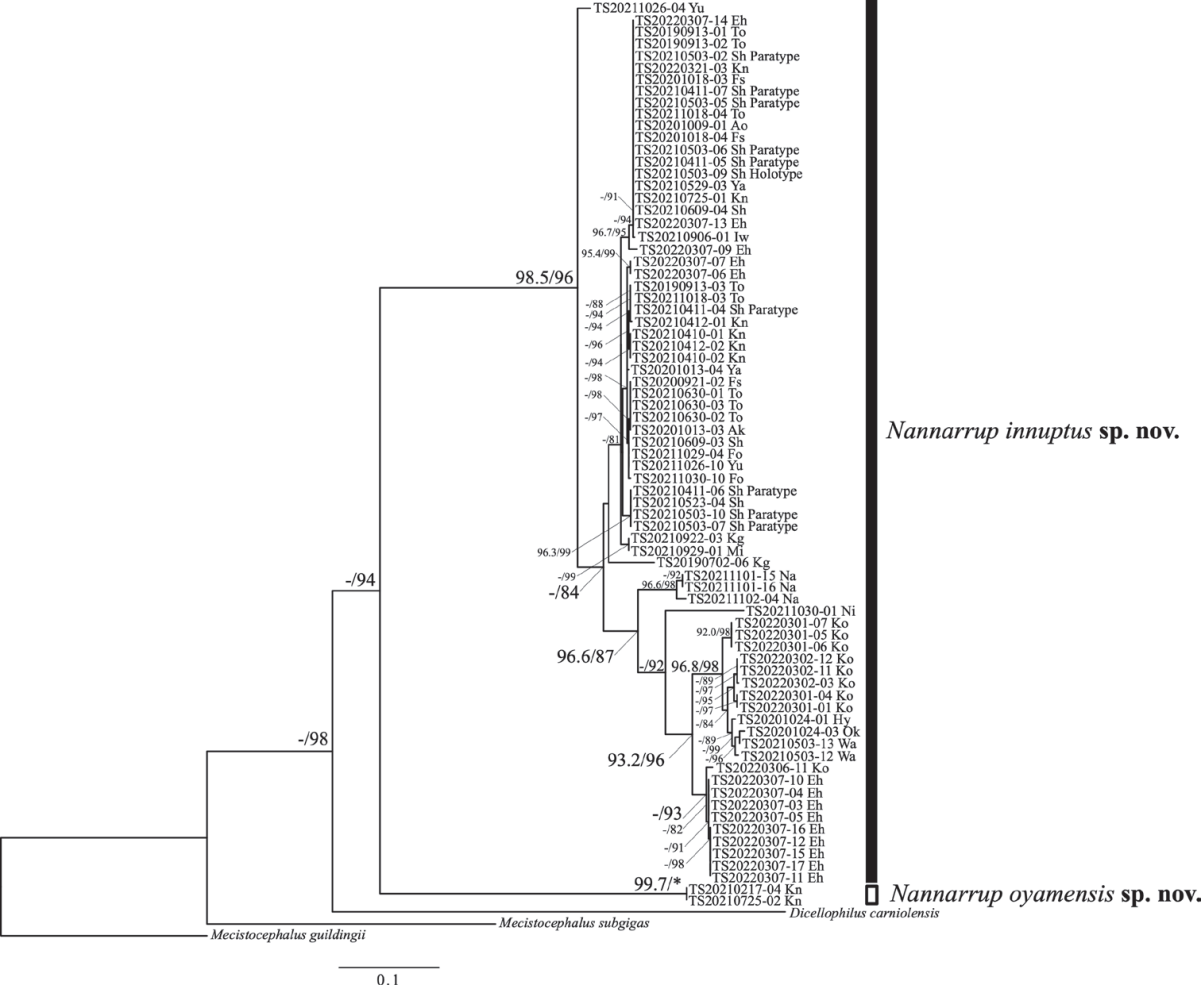
Maximum-likelihood tree of *Nannarrup* specimens and outgroups (*Mecistocephalusguildingii*, *M.subgigas*, and *Dicellophiluscarniolensis*) based on the *COI* dataset. Nodal values are derived from Ultrafast bootstrap (UFBoot) and SH-like approximate likelihood ratio test (SH-aLRT). Asterisk (*) indicates values of 100% in UFBoot and SH-aLRT. A hyphen (-) indicates values <95% in UFBoot and <80% in SH-aLRT. Nodal values are not shown when both UFBoot and SH-aLRT values are <95% and <80%, respectively. The unit of evolutionary distance is the number of base substitutions per site. Ingroup specimens are shown as their specimen identification number. Abbreviations: Ao = Aomori pref.; Ak = Akita pref.; Iw = Iwate pref.; Ya = Yamagata pref.; Fs = Fukushima pref.; Ni = Niigata pref.; To = Tokyo pref.; Kn = Kanagawa pref.; Sh = Shizuoka pref.; Wa = Wakayama pref.; Hy = Hyogo pref.; Ok = Okayama pref.; Yu = Yamaguchi pref.; Ko = Kochi pref.; Eh = Ehime pref.; Fo = Fukuoka pref.; Mi = Miyazaki pref.; Kg = Kagoshima pref.

In the ML phylogenetic trees based on the *COI* dataset (Fig. 2), the clade comprising *N.* sp. 1 and *N.* sp. 2 was moderately supported (UFBoot = 81.2%, SH-aLRT = 94%). However, *N.* sp. 1 and *N.* sp. 2 were distinctly separated from each other, and each morphospecies was strongly supported in monophyly (UFBoot = 98.5%, SH-aLRT = 96% in *N.* sp. 1; UFBoot = 99.7%, SH-aLRT = 100% in *N.* sp. 2). The intraspecific phylogeographic structure of *N.* sp. 1 remains unclear because of low support values. It is noteworthy that one specimen, TS20210725-01, had been collected near the collection site of *N.* sp. 2, but this specimen was included in the *N.* sp. 1 clade (see also Fig. 1).

**Figure 3.**
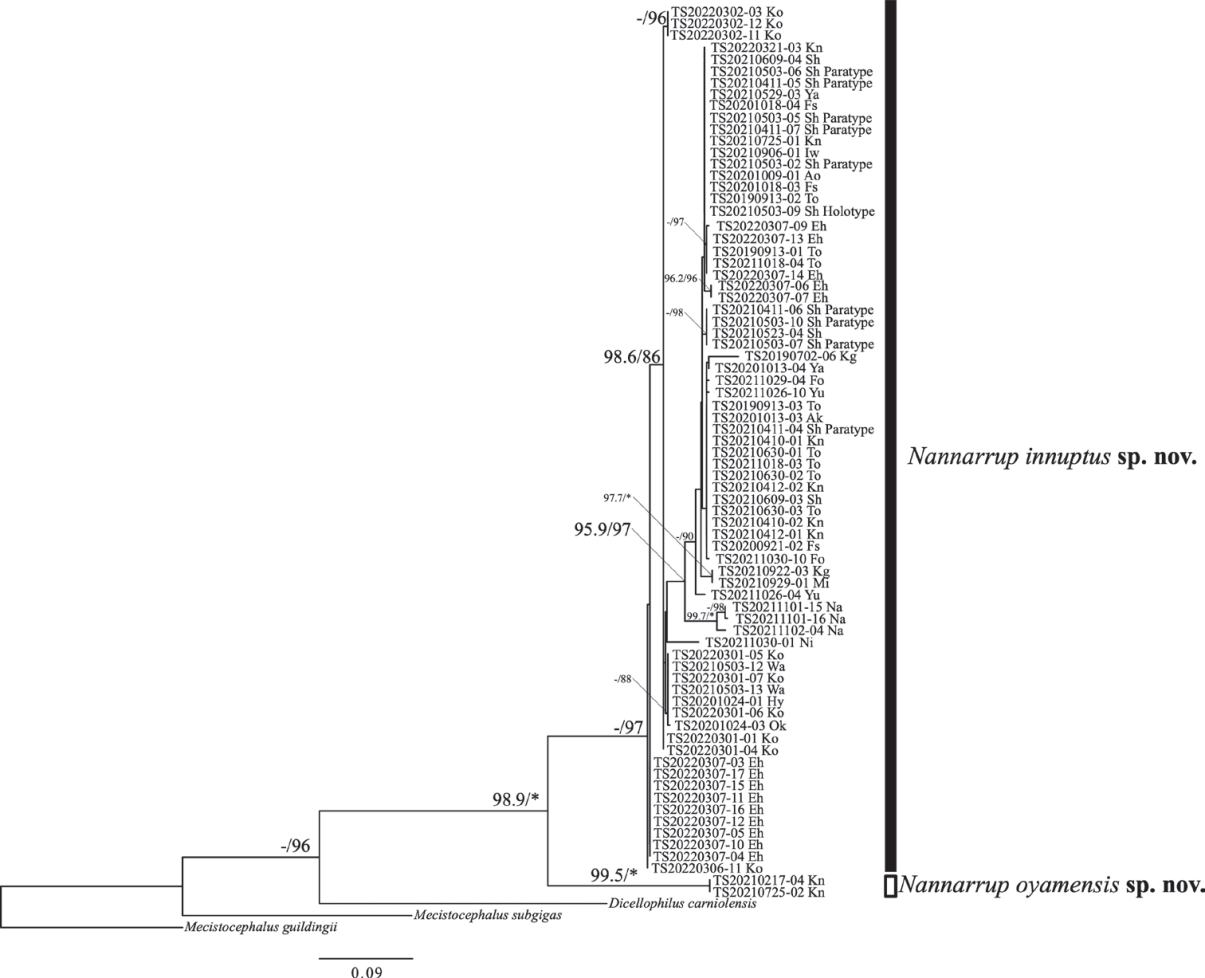
Maximum-likelihood tree of *Nannarrup* specimens and outgroups (*Mecistocephalusguildingii*, *M.subgigas*, and *Dicellophiluscarniolensis*) based on the *16S* dataset. Nodal values are derived from Ultrafast bootstrap (UFBoot) and SH-like approximate likelihood ratio test (SH-aLRT). Asterisk (*) indicates values of 100% in UFBoot and SH-aLRT. A hyphen (-) shows < 95% in UFBoot and < 80% in SH-aLRT. Nodal values are not shown when both UFBoot and SH-aLRT values are < 95% and < 80%, respectively. The unit of evolutionary distance is the number of base substitutions per site. Ingroup specimens are shown as their specimen identification number. Abbreviations: Ao = Aomori pref.; Ak = Akita pref.; Iw = Iwate pref.; Ya = Yamagata pref.; Fs = Fukushima pref.; Ni = Niigata pref.; To = Tokyo pref.; Kn = Kanagawa pref.; Sh = Shizuoka pref.; Wa = Wakayama pref.; Hy = Hyogo pref.; Ok = Okayama pref.; Yu = Yamaguchi pref.; Ko = Kochi pref.; Eh = Ehime pref.; Fo = Fukuoka pref.; Mi = Miyazaki pref.; Kg = Kagoshima pref.

The ML phylogenetic trees based on the *16S* dataset (Fig. 3) showed that the clade comprising *N.* sp. 1 and *N.* sp. 2 was highly supported (UFBoot = 98.9%, SH-aLRT = 100%), and *N.* sp. 1 and *N.* sp. 2 were also distinctly separated from each other. Each morphospecies was also strongly supported in their monophyly, as observed in the phylogenetic trees based on the *COI* dataset (UFBoot = 92.4%, SH-aLRT = 97% in *N.* sp. 1; UFBoot = 99.5%, SH-aLRT = 100% in *N.* sp. 2). The intraspecific phylogeographic structure of *N.* sp. 1 remains obscure because of low support values. TS20210725-01 is included in the *N.* sp. 1 clade as seen in the *COI* trees.

**Figure 4.**
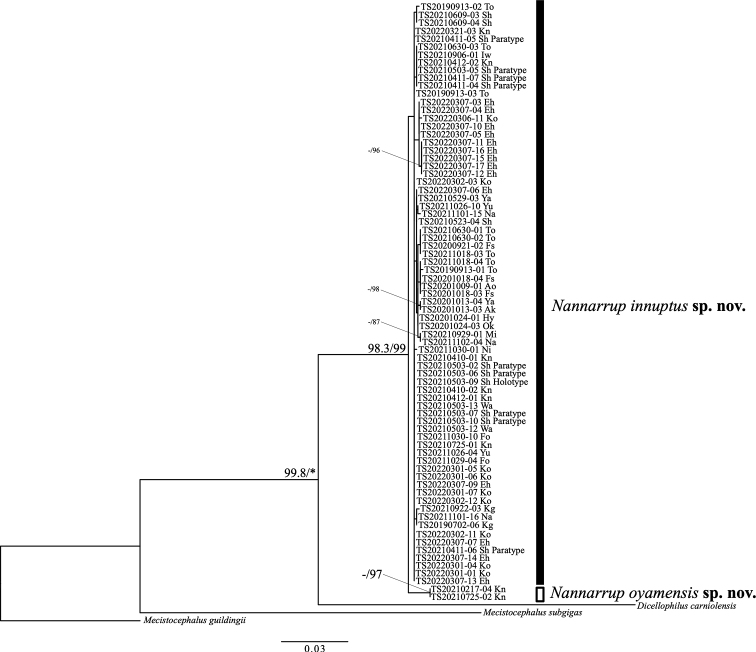
Maximum-likelihood tree of *Nannarrup* specimens and outgroups (*Mecistocephalusguildingii*, *M.subgigas*, and *Dicellophiluscarniolensis*) based on the *28S* dataset. Nodal values are derived from Ultrafast bootstrap (UFBoot) and SH-like approximate likelihood ratio test (SH-aLRT). Asterisk (*) indicates values of 100% in UFBoot and SH-aLRT. A hyphen (-) shows < 95% in UFBoot and < 80% in SH-aLRT. Nodal values are not shown when both UFBoot and SH-aLRT values are < 95% and < 80%, respectively. The unit of evolutionary distance is the number of base substitutions per site. Ingroup specimens are shown as their specimen identification number. Abbreviations: Ao = Aomori pref.; Ak = Akita pref.; Iw = Iwate pref.; Ya = Yamagata pref.; Fs = Fukushima pref.; Ni = Niigata pref.; To = Tokyo pref.; Kn = Kanagawa pref.; Sh = Shizuoka pref.; Wa = Wakayama pref.; Hy = Hyogo pref.; Ok = Okayama pref.; Yu = Yamaguchi pref.; Ko = Kochi pref.; Eh = Ehime pref.; Fo = Fukuoka pref.; Mi = Miyazaki pref.; Kg = Kagoshima pref.

In the ML phylogenetic trees based on the *28S* dataset (Fig. 4), the clade comprising *N.* sp. 1 and *N.* sp. 2 was strongly supported (UFBoot = 98.3%, SH-aLRT = 99%). The monophyly of *N.* sp. 1 was not well supported (UFBoot = 62.6%, SH-aLRT = 49%), but the monophyly of *N.* sp. 2 was moderately supported (UFBoot = 91.7%, SH-aLRT = 97%).

### ﻿DNA barcoding of the Japanese *Nannarrup* specimens

According to the *COI* dataset of *Nannarrup*, the minimum divergence between *N.* sp. 1 and *N.* sp. 2 was 14.13% in p-distance and 15.73% in K2P distance (TS20211101-15 and TS20211101-16 from Nagasaki prefecture vs TS20210217-04 and TS20210725-02 from Kanagawa prefecture), whereas the maximum internal divergence within *N.* sp. 1 was 9.726% in p-distance and 10.58% in K2P distance (TS20211030-01 from Niigata prefecture vs TS20210412-01 from Kanagawa prefecture).

According to the *16S* dataset of *Nannarrup*, the minimum divergence between *N.* sp. 1 and *N.* sp. 2 was 14.92% in p-distance and 16.68% in K2P distance (TS20220306-11 from Kochi prefecture and TS20220307-03, TS20220307-04, TS20220307-05, TS20220307-11, TS20220307-12, TS20220307-15, TS20220307-16, TS20220307-17 from Ehime prefecture vs TS20210217-04 and TS20210725-02 from Kanagawa prefecture), whereas the maximum internal divergence within *N.* sp. 1 was 7.056% in p-distance and 7.443% in K2P distance (TS20211030-01 from Niigata prefecture vs TS20211102-04 from Nagasaki prefecture).

According to the *28S* dataset of *Nannarrup*, the minimum divergence between *N.* sp. 1 and *N.* sp. 2 was 1.207% in p-distance and 1.218% in K2P distance (TS20220301-01, TS20220301-04 TS20220302-03, TS20220302-11 from Kochi prefecture, TS20220307-07, TS20220307-13, TS20220307-14 from Ehime prefecture and TS20200411-06 from Shizuoka prefecture vs TS20210217-04 and TS20210725-02 from Kanagawa prefecture), whereas the maximum internal divergence within *N.* sp. 1 was 0.6757% in p-distance and 0.6796% in K2P distance (TS20220307-11, TS20220307-12, TS20220307-15, TS20220307-16, TS20220307-17 from Ehime prefecture vs TS20190913-01 from Tokyo prefecture).

### ﻿Taxonomic account

#### Family Mecistocephalidae Bollmann, 1893

##### ﻿Genus Nannarrup

Taxon classificationAnimaliaGeophilomorphaMecistocephalidae

Foddai, Bonato, Pereira & Minelli, 2003

6BE9A183-A5D3-576B-AFCD-9B2782EB3610

**Figure 5.**
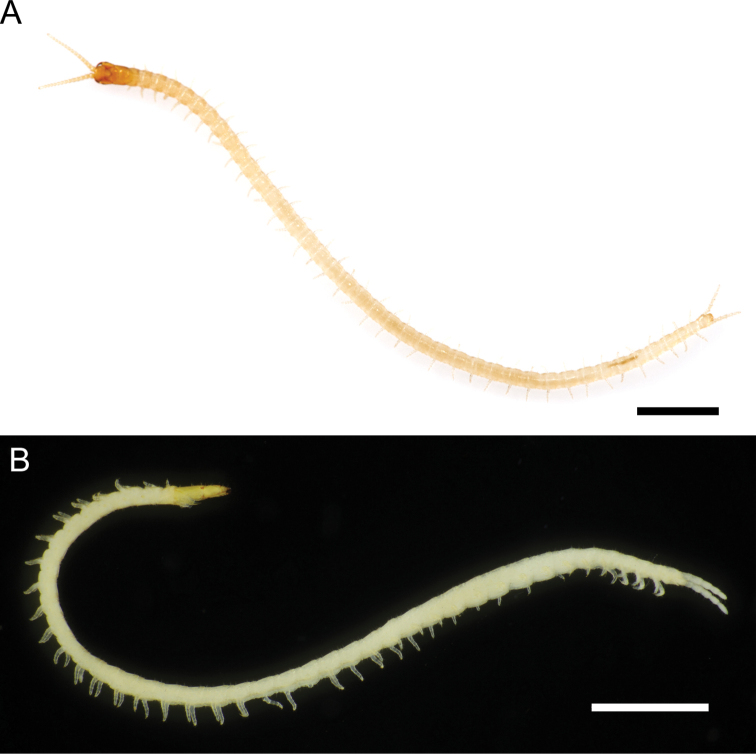
*Nannarrupinnuptus* sp. nov., paratype (TS20210503-02) **A** habitus (provided by Dr Namiki Kikuchi) **B** “death pose” with head capsule detached. Scale bars: 1 mm.

New Japanese name: Himejimukade-zoku Fig. 5

Nannarrup Foddai, Bonato, Pereira & Minelli, 2003: 1255–1256.

###### Type species.

*Nannarruphoffmani* Foddai, Bonato, Pereira & Minelli, 2003

###### Diagnosis.

Partly modified from Foddai et al. (2003). Adult body length ca 10 mm (Fig. 5A). Cephalic plate only slightly longer than wide, with frontal line absent or replaced by areolation. Two small clypeal plagulae covering ca one-sixth of the clypeus. Bucca without setae. Stilus present, relatively short. Spiculum absent. Side-pieces of labrum only incompletely subdivided into anterior and posterior alae by fragmented line very poorly marked. Mandible provided with four well-developed pectinate lamellae. Coxosternite of first maxillae medially divided. Coxosternite of second maxillae undivided, without suture or membranous isthmus. Metameric pore close to posterior margin of coxosternum of second maxillae, not to lateral ones. Claw of second maxillae only represented by terminal spine. Forcipular telopodites far behind anterior margin of head in the closed position. Forcipular trochanteroprefemur with pigmented single distal denticle; femur without teeth; tarsungulum with basal, well-developed denticle. Forcipular tergite without median sulcus. Sternal sulcus not anteriorly furcate. Last metasternite subtriangular. Ventral surface of each coxopleuron with numerous pores. Anal pore present. Forty-one pairs of legs.

###### Remarks.

The following characters included in the diagnosis sensu Foddai et al. (2003) are different among the three *Nannarrup* species: presence/absence of a pair of smooth or areolate areas along the posterior part of the paraclypeal sutures, presence/absence of a tubercle on the forcipular tibia, pigmentation of the denticle on the tarsungulum (Table 3). In total, 74 out of 88 collected specimens of *Nannarrup* (ca 84%) exhibited a leaning posture or even threw back their head when stored in ethanol (Fig. 5B). Although such a “death pose” has not been quantitatively investigated in Geophilomorpha, in the authors’ experience, it is a unique phenomenon in *Nannarrup* that may be related to the internal morphological characteristics of the genus.

##### ﻿ Nannarrup innuptus

Taxon classificationAnimaliaGeophilomorphaMecistocephalidae

Tsukamoto sp. nov.

6440CA97-9C1E-52D8-B388-2368A02A34F9

https://zoobank.org/D2906856-517E-45BE-B2A0-1FE9CBD4D779

**Figure 6.**
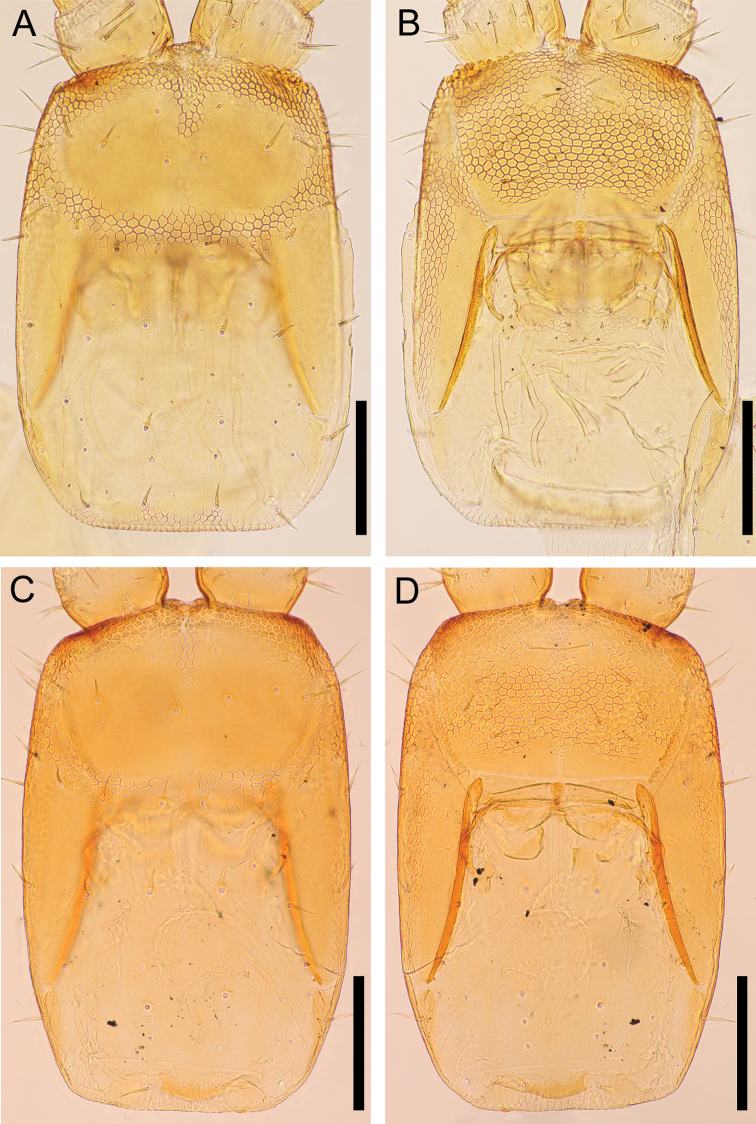
*Nannarrupinnuptus* sp. nov. **A, B** holotype (TS20210503-09) **C, D** paratype (TS20210411-04). **A, B** cephalic plate, dorsal **C, D** clypeus and clypeal pleurite, ventral. Scale bars: 0.3 mm.

**Figure 7.**
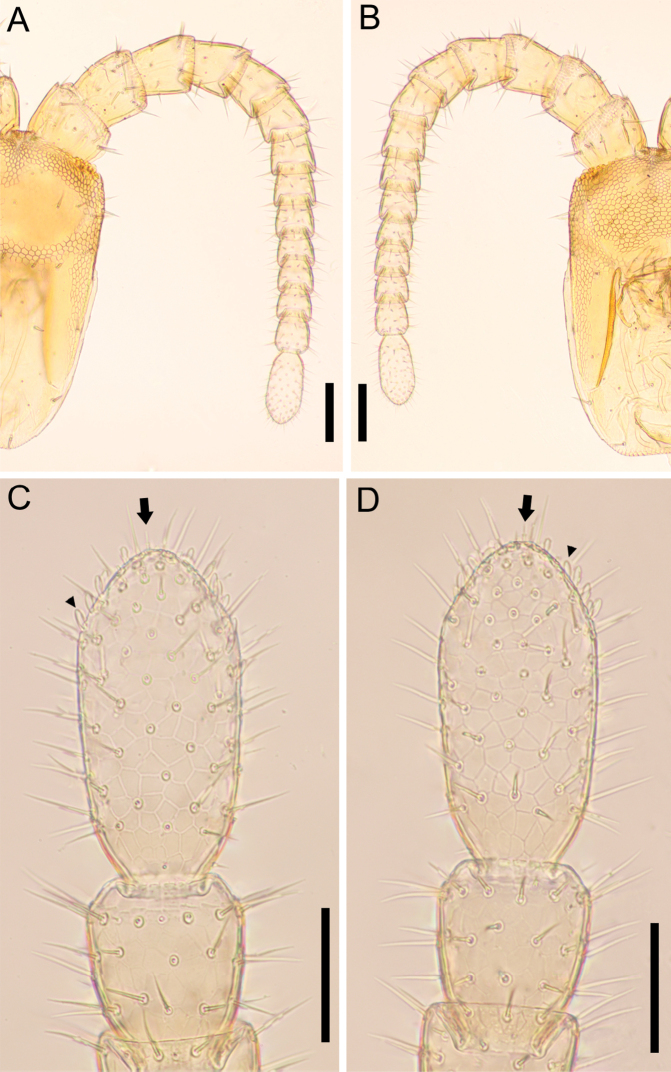
*Nannarrupinnuptus* sp. nov., holotype (TS20210503-09) **A** right part of head and right antenna, dorsal **B** right part of head and right antenna, ventral **C** antennal article XIV, dorsal **D** antennal article XIV, ventral. Arrows indicate apical sensillum; arrowheads indicate club-like sensillum. Scale bars: 0.1 mm (**A, B**); 0.05 mm (**C, D**).

**Figure 8.**
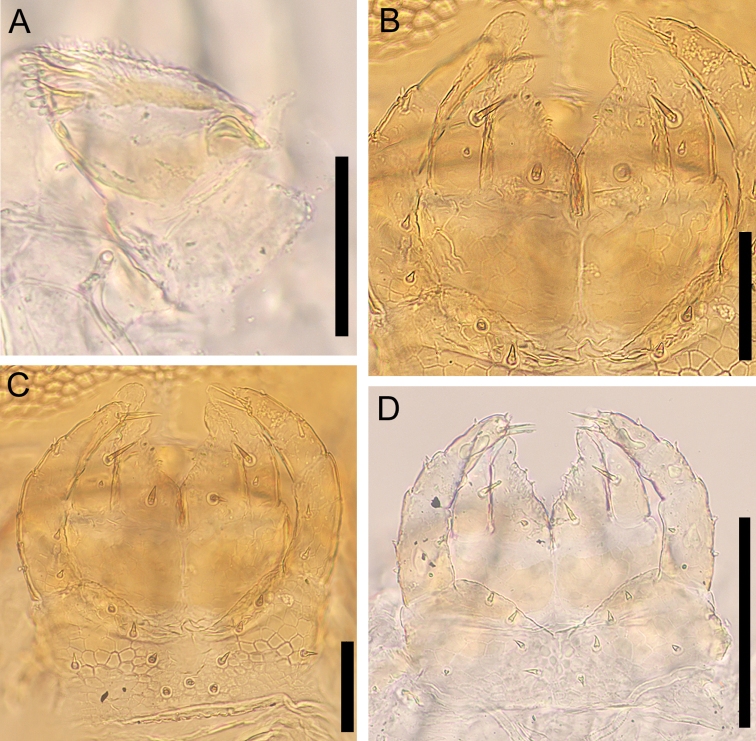
*Nannarrupinnuptus* sp. nov. **A, D** paratype (TS20210411-05) **B, C** holotype (TS20210503-09). **A** right mandible, dorsal **B** first maxillae, ventral **C, D** second maxillae, ventral. Scale bars: 0.05 mm.

**Figure 9.**
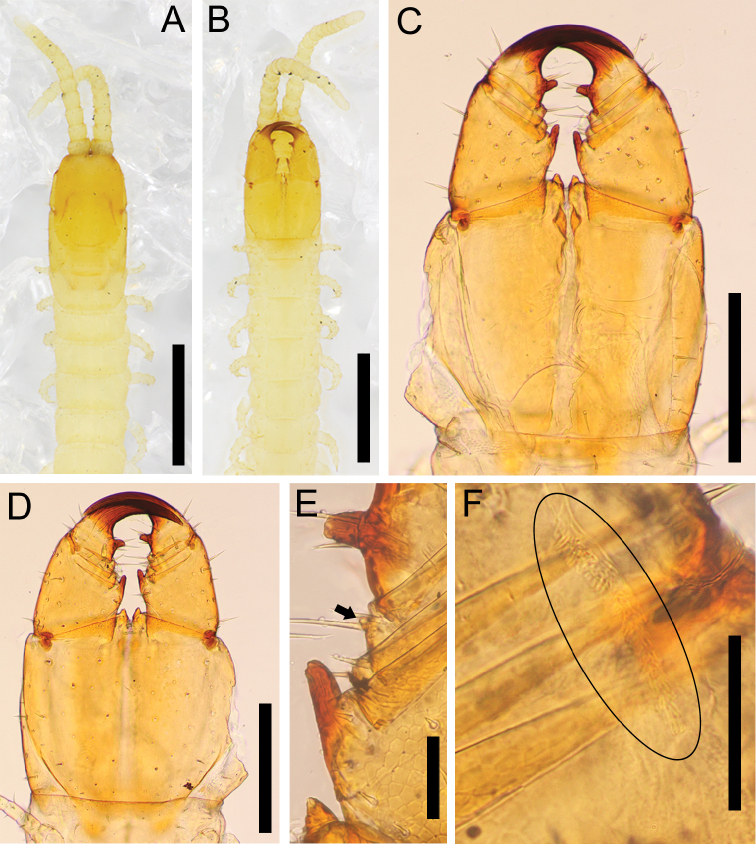
*Nannarrupinnuptus* sp. nov. **A, B** paratype (TS20210411-05) **C, D** holotype (TS20210503-09) **E, F** paratype (TS20210411-04). **A** anterior part of body, dorsal **B** anterior part of body, ventral **C** forcipular segment, dorsal **D** forcipular segment, ventral **E** denticles on forcipule, dorsal **F** poison calyx, dorsal. Arrow indicates tubercle on tibia. Circle indicates poison calyx. Scale bars: 0.5 mm (**A, B**); 0.3 mm (**C, D**); 0.05 mm (**E, F**).

**Figure 10.**
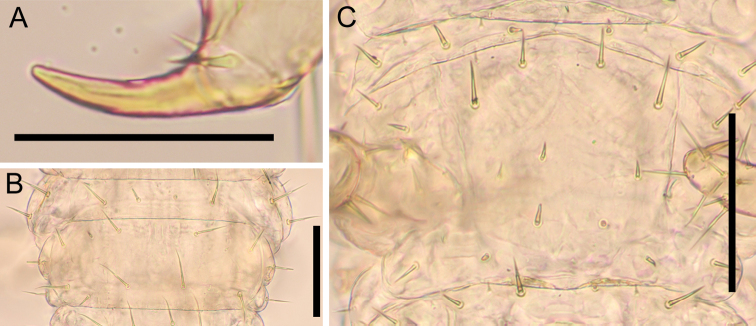
*Nannarrupinnuptus* sp. nov. **A** holotype (TS20210503-09) **B, C** paratype (TS20210411-05) **A** pretarsus of left leg 2, anterolateral **B** tergite of leg-bearing segment 40, dorsal **C** sternite of leg-bearing segment 40, ventral. Scale bars: 0.05 mm (**A**); 0.1 mm (**B, C**).

**Figure 11.**
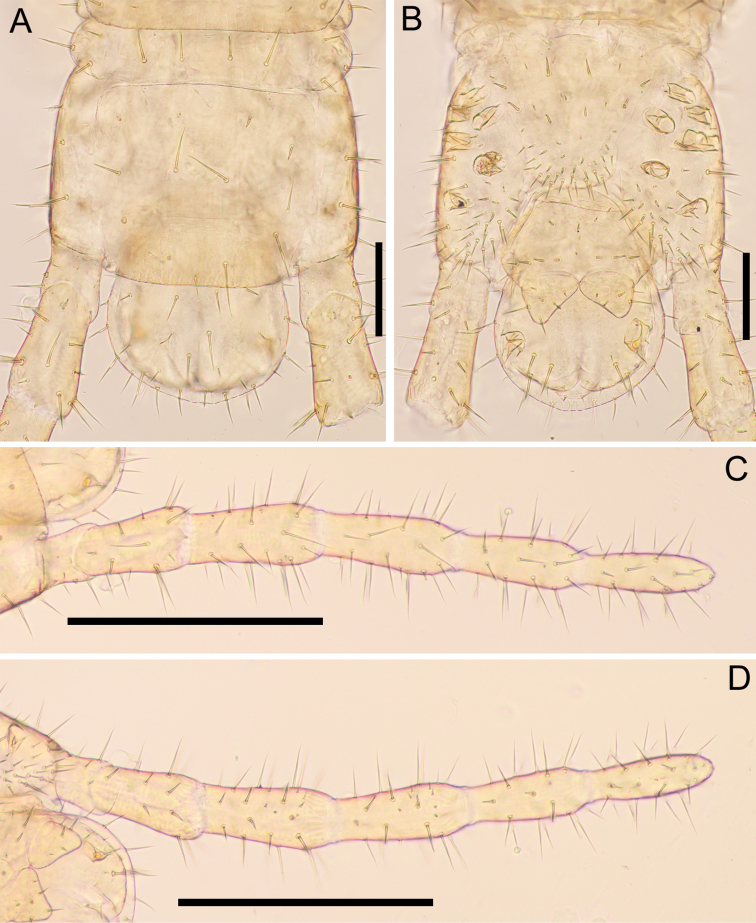
*Nannarrupinnuptus* sp. nov., holotype (TS20210503-09) **A** ultimate leg-bearing segment and postpedal segment, dorsal **B** ultimate leg-bearing segment and postpedal segment, ventral **C** left ultimate leg, dorsal **D** left ultimate leg, ventral. Scale bars: 0.1 mm (**A, B**); 0.3 mm (**C, D**).

New Japanese name: Kaguya-himejimukade Figs 5 , 6 , 7 , 8 , 9 , 10 , 11

###### Type material.

***Holotype*.** 1 adult female, Yugashima, Izu-shi, Shizuoka prefecture, Japan (34°51.39'N, 138°55.40'E), 3 May 2021, coll. Mayu Susukida (labeled as TS20210503-09), deposited at the Collection of Myriapoda, Department of Zoology, **NSMT**.

***Paratype*.** 4 females, Yugashima, Izu-shi, Shizuoka prefecture, Japan (34°51.39'N, 138°55.39'E), 11 April 2021, leg. Katsuyuki Eguchi (labeled as TS20210411-04, TS20210411-05, TS20210411-06, TS20210411-07, respectively), deposited at the Collection of Myriapoda, Department of Zoology, **NSMT**. 5 females, Yugashima, Izu-shi, Shizuoka prefecture, Japan (34°51.39'N, 138°55.40'E), 3 May 2021, leg. Mayu Susukida (labeled as TS20210503-02, TS20210503-05, TS20210503-06, TS20210503-07, TS20210503-10, respectively), deposited at **MNHAH**.

###### Non-type specimens.

1 female, Minamiosawa, Hachioji-shi, Tokyo prefecture, Japan (35°37.02'N, 139°22.73'E), 27 June 2018, leg. Sho Tsukamoto (labeled as TS20180627-01). 1 female, Hirasawa, Akiruno-shi, Tokyo prefecture, Japan (35°43.64'N, 139°19.20'E), 10 October 2017, leg. Sho Tsukamoto (labeled as TS20171010-01). 3 females, Hirasawa, Akiruno-shi, Tokyo prefecture, Japan (35°43.64'N, 139°19.20'E), 13 September 2019, leg. Sho Tsukamoto (labeled as TS20190913-01, TS20190913-02, TS20190913-03, respectively). 1 female, Shiroyama, Kagoshima-shi, Kagoshima prefecture, Japan (31°35.88'N, 130°32.98'E), 2 July 2019, leg. Sho Tsukamoto (labeled as TS20190702-06). 1 female, Shibakusa, Hatori, Ten-ei-mura, Iwase-gun, Fukushima prefecture, Japan (37°14.37'N, 140°03.86'E), 21 September 2020, leg. Katsuyuki Eguchi (labeled as TS20200921-02). 1 female, Kubo, Hiranuma, Rokkasho-mura, Kamikita-gun, Aomori prefecture, Japan (40°52.37'N, 141°21.76'E), 9 October 2020, leg. Katsuyuki Eguchi (labeled as TS20201009-01). 1 female, Nakagawara, Nagano, Daisen-shi, Akita prefecture, Japan (39°32.41'N, 140°31.76'E), 13 October 2020, leg. Katsuyuki Eguchi (labeled as TS20201013-03). 1 female, Mukounadaka, Nadaka, Tozawa-mura, Mogami-gun, Yamagata prefecture, Japan (38°44.96'N, 140°11.15'E), 13 October 2020, leg. Katsuyuki Eguchi (labeled as TS20201013-04). 1 female, Nakagawa, Kaneyama-machi, Onuma-gun, Fukushima prefecture, Japan (37°28.25'N, 139°31.81'E), 18 October 2020, leg. Katsuyuki Eguchi (labeled as TS20201018-03). 1 female, Sawanishi, Mizunuma, Kaneyama-machi, Onuma-gun, Fukushima prefecture, Japan (37°28.87'N, 139°33.48'E), 18 October 2020, leg. Katsuyuki Eguchi (labeled as TS20201018-04). 1 female, Tai, Yamasaki-cho, Shisou-shi, Hyogo prefecture, Japan (35°02.62'N, 134°33.68'E), 24 October 2020, leg. Katsuyuki Eguchi (labeled as TS20201024-01). 1 female, Kageishi, Nishiawakura-son, Aida-gun, Okayama prefecture, Japan (35°10.94'N, 134°20.64'E), 24 October 2020, leg. Katsuyuki Eguchi (labeled as TS20201024-03). 2 females and 2 juveniles (sex unknown), Teraodai, Ayase-shi, Kanagawa prefecture, Japan (35°27.76'N, 139°25.13'E), 10 April 2021, leg. Joe Kutsukake (labeled as TS20210410-01 and TS20210410-02 for females, TS20210410-03 and TS20210410-04 for juveniles, respectively). 2 females, Nebukawa, Odawara-shi, Kanagawa prefecture, Japan (35°12.00'N, 139°08.22'E), 12 April 2021, leg. Joe Kutsukake (labeled as TS20210412-01 and TS20210412-02, respectively). 3 juveniles (sex unknown), Nebukawa, Odawara-shi, Kanagawa prefecture, Japan (35°12.23'N, 139°08.43'E), 12 April 2021, leg. Joe Kutsukake (labeled as TS20210412-03, TS20210412-04 and TS20210412-05, respectively). 1 female, Shimada, Inami-cho, Hidaka-gun, Wakayama prefecture, Japan (33°47.36'N, 135°14.06'E), 3 of May 2021, leg. Katsuyuki Eguchi (labeled as TS20210503-12). 1 female, Kurisugawa, Nakahechi-cho, Tanabe-shi, Wakayama prefecture, Japan (33°47.69'N, 135°30.16'E), 3 of May 2021, leg. Katsuyuki Eguchi (labeled as TS20210503-13). 1 female, Futo, Ito-shi, Shizuoka prefecture, Japan (34°54.58'N, 139°07.72'E), 9 June 2021, leg. Joe Kutsukake (labeled as TS20210609-03). 1 female, Futo, Ito-shi, Shizuoka prefecture, Japan (34°54.66'N, 139°07.19'E), 9 June 2021, leg. Joe Kutsukake (labeled as TS20210609-04). 1 juvenile (sex unknown), Shishihara, Shimizu-ku, Shizuoka-shi, Shizuoka prefecture, Japan (35°11.94'N, 138°31.28'E), 23 of May 2021, leg. Katsuyuki Eguchi (labeled as TS20210523-04). 1 female, Nishiaraya, Tsuruoka-shi, Yamagata prefecture, Japan (38°38.64'N, 139°49.73'E), 29 May 2021, leg. Katsuyuki Eguchi (labeled as TS20210529-03). 3 females, Den-enchofu, Ota-ku, Tokyo prefecture, Japan (35°35.51'N, 139°39.86'E), 30 June 2021, leg. Joe Kutsukake (labeled as TS20210630-01, TS20210630-02 and TS20210630-03). 1 subadult female, Oyama, Isehara-shi, Kanagawa prefecture, Japan (35°25.74'N, 139°14.44'E), 25 July 2021, coll. Sho Tsukamoto (labeled as TS20210725-01; cephalic capsule lost). 1 female, Hikime, Miyako-shi, Iwate prefecture, Japan (39°37.49'N, 141°49.41'E), 6 September 2021, leg. Katsuyuki Eguchi (labeled as TS20210906-01). 3 juveniles (sex unknown), Hiyamizucho, Kagoshima-shi, Kagoshima prefecture, Japan (31°36.21'N, 130°33.02'E), 22 September 2021, leg. Joe Kutsukake (labeled as TS20210922-01, TS20210922-02 and TS20210922-03). 1 female, Natsuocho, Miyakonojo-shi, Miyazaki prefecture, Japan (31°52.49'N, 130°57.50'E), 29 September 2021, leg. Joe Kutsukake (labeled as TS20210929-01). 2 females and 1 juvenile (sex unknown), Minamiosawa, Hachioji-shi, Tokyo prefecture, Japan (35°37.43'N, 139°23.05'E), 18 October 2021, leg. Joe Kutsukake (labeled as TS20211018-03, TS20211018-04 for females and TS20211018-05 for the juvenile, respectively). 2 females and 1 juvenile (sex unknown), Era, Toyotacho, Shimonoseki-shi, Yamaguchi prefecture, Japan (34°10.56'N, 131°02.48'E), 26 October 2021, leg. Sho Tsukamoto (labeled as TS20211026-04, TS20211026-10 for females and TS20211026-05 for the juvenile, respectively). 1 female, Hikosan, Soeda-machi, Tagawa-gun, Fukuoka prefecture, Japan (33°29.06'N, 130°55.94'E), 29 October 2021, leg. Sho Tsukamoto (labeled as TS20211029-04). 1 female, Tomaruhinoe, Tsunan-machi, Nakauonuma-gun, Niigata prefecture, Japan (37°02.16'N, 138°39.46'E), 30 October 2021, leg. Katsuyuki Eguchi (labeled as TS20211030-01). 1 female, Maeda, Yahatahigashi-ku, Kitakyushu-shi, Fukuoka prefecture, Japan (33°51.35'N, 130°47.72'E), 30 October 2021, leg. Sho Tsukamoto (labeled as TS20211030-10). 2 females and 2 specimens (the lower half of body lost, sex unknown), Nishiyama, Nagasaki-shi, Nagasaki prefecture, Japan (32°45.89'N, 129°53.00'E), 1 November 2021, leg. Sho Tsukamoto (labeled as TS20211101-15, TS20211101-16 for females and TS20211101-17, TS20211101-18 for sex-unknown specimens, respectively). 1 female, Nijigaoka, Nagasaki-shi, Nagasaki prefecture, Japan (32°47.73'N, 129°50.00'E), 2 November 2021, leg. Sho Tsukamoto (labeled as TS20211102-04). 1 female, Murotsu, Muroto-shi, Kochi prefecture, Japan (33°18.06'N, 134°09.31E), 1 March 2022, leg. Katsuyuki Eguchi (labeled as TS20220301-01). 1 female, Murotomisakicho, Muroto-shi, Kochi prefecture, Japan (33°16.87'N, 134°10.65E), 2 March 2022, leg. Joe Kutsukake (labeled as TS20220302-03). 1 female, Kamoi, Yokosuka-shi, Kanagawa prefecture, Japan (35°15.44'N, 139°44.61E), 21 March 2022, leg. Katsuyuki Eguchi (labeled as TS20220321-03). 1 female, Murotsu, Muroto-shi, Kochi prefecture, Japan (33°18.07'N, 134°09.31E), 1 March 2022, leg. Joe Kutsukake (labeled as TS20220301-04). 3 females, Makigawa, Tsushimacho, Uwajima-shi, Ehime prefecture, Japan (33°05.74'N, 132°35.58E), 7 March 2022, leg. Joe Kutsukake (labeled as TS20220307-03, TS20220307-04, TS20220307-05, respectively). 2 females, Sunokawa, Ainan-cho, Minamiuwa-gun, Ehime prefecture, Japan (33°02.50'N, 132°29.18E), 7 March 2022, leg. Joe Kutsukake (labeled as TS20220307-06, TS20220307-07, respectively). 1 female, Matsuo, Tosashimizu-shi, Kochi prefecture, Japan (32°44.16'N, 132°58.57E), 6 March 2022, leg. Joe Kutsukake (labeled as TS20220306-11). 2 females, Makigawa, Tsushimacho, Uwajima-shi, Ehime prefecture, Japan (33°05.87'N, 132°36.98E), 7 March 2022, leg. Joe Kutsukake (labeled as TS20220307-09, TS20220307-10, respectively). 2 females, Ryoke, Muroto-shi, Kochi prefecture, Japan (33°17.23'N, 134°10.59E), 2 March 2022, leg. Joe Kutsukake (labeled as TS20220302-11, TS20220302-12, respectively). 2 females and 1 specimen (the lower half of body lost, sex unknown), Motootsu, Muroto-shi, Kochi prefecture, Japan (33°18.81'N, 134°07.33E), 1 March 2022, leg. Joe Kutsukake (labeled as TS20220301-05, TS20220301-06 for females and TS20220301-07 for sex-unknown specimens, respectively). 7 females, Iwabuchi, Tsushimacho, Uwajima-shi, Ehime prefecture, Japan (33°08.81'N, 132°32.99E), 7 March 2022, leg. Joe Kutsukake (labeled as TS20220307-11, TS20220307-12, TS20220307-13, TS20220307-14, TS20220307-15, TS20220307-16, TS20220307-17, respectively).

###### Etymology.

The species name is derived from unmarried in Latin. In the Japanese population, males of this species have not been discovered despite the wide collection range in Japan.

###### Diagnosis.

Clypeus with a pair of smooth or weakly areolate areas along the posterior part of the paraclypeal sutures; forcipular trochantroprefemur with a large denticle (longer than wide); tarsungulum with a well-pigmented denticle; metasternite of ultimate leg-bearing segment wider than long.

###### Description.

***General features*** (Fig. 5A, B): Body 7.0–12.0 mm long (holotype 12.0 mm), gradually attenuated posteriorly, almost uniformly very pale yellow, with head and forcipular segment pale ocher.

***Cephalic capsule*** (Fig. 6A–D): Cephalic plate ca 1.4–1.6× as long as wide; lateral margins more distinctly converging anteriorly than posteriorly; posterior margin straight; scutes approximately isometric and up to 15 μm wide; transverse suture absent but areolate line present in some individuals (Fig. 6A, C); setae up to ca 37.5 μm long. Clypeus ca 1.4–1.5× as wide as long, with lateral margins complete, almost uniformly areolate, with scutes ca 15 μm wide, clypeal areas absent; clypeus with 11–17 setae, 1+1 postantennal, 1–2+2 median, 3–6+3–5 prelabral; clypeal ratio ca 1: 6–1: 7; clypeal plagulae with additional smooth or weak areolation area along posterior part of paraclypeal sutures. Anterior and distolateral parts of pleurites areolate, without setae. Side-pieces of labrum medially in contact, only incompletely divided into anterior and posterior alae by weak chitinous line, without longitudinal stripes on posterior alae, with slightly visible short fringe on posterior margin of side-pieces; mid-piece as long as wide, converging anteriorly and posteriorly.

***Antenna*** (Fig. 7A–D): Antenna with 14 articles, when stretched, ca 2.1–2.6× as long as head length. Intermediate articles slightly longer than wide. Article XIV ca 2.5× as long as wide, ca 1.9–2.4× as long as article XIII, and 1.8–2.4× as long as intermediate articles. Setae on articles VIII–XVI denser than articles I–VII. Setae gradually shorter from article VIII to XIV, up to 65 μm long on article I, up to 25 µm long on article VIII and < 15 μm long on article XIV. Article XIV with two types of sensilla; apical sensilla (arrows in Fig. 7C, D) ca 10 μm long, with wide flat ring at mid-length; club-like (arrowheads in Fig. 7C, D) sensilla ca 15 μm long, clustered in the distal part of the internal and the external sides of the article. Three longitudinal rows, each consists of ca 9 proprioceptive spine-like sensilla, at bases of antennal articles III–V, VII–IX, approximately dorsal, ventro-internal and ventro-external; the rows each consisting of 1–3 spine-like sensilla on articles I and VI, and 6 on the article II; 0–1 spine-like sensilla on articles X–XIV. A few pointed sensilla, up to 2.5 μm long, on both dorso-external and ventro-internal position, close to distal margin of articles II, V, IX and XIII.

***Mandible*** (Fig. 8A): At least four pectinate lamellae present; first and second lamellae with ca 5 elongated teeth. Each tooth ca 2× as long as wide. Ventral surface hairy.

***First maxillae*** (Fig. 8B): Coxosternite medially divided but slightly, without setae, faintly areolate. Coxal projections well developed and hyaline distally, with 1–2+1–2 setae and 3–4+3–4 small sensilla. Telopodite uni-articulated and hyaline distally, with one(two) seta(e). No lobes on either coxosternite or telopodites.

***Second maxillae*** (Fig. 8C, D): Coxosternite medially undivided, without suture, with 2+2 setae along anterior margin, with 6–7 setae behind anterior margin, with 1–2+1–2 sensilla on posterior lateral margin in some individuals. with anterior margin slightly concave, with metameric pores on posterior part. Telopodites tri-articulate, reaching medial projections and telopodites of first maxillae in some individuals. Claw of telopodite virtually absent, represented by short spine only.

***Forcipular segment*** (Fig. 9A–F): Tergite trapezoidal, ca 1.4–1.9× as wide as long, with lateral margins converging anteriorly, approximately as wide as cephalic plate and ca 0.7× as wide as following tergites; 1+1 setae of similar length arranged in an anterior row, and 3+3 setae of similar length arranged in a posterior row; one pair of longitudinal rows of three tiny setae located between middle and distal setae in posterior row. Mid-longitudinal sulcus of tergite not visible. Exposed part of coxosternite ca 1.1× as wide as long; anterior margin with shallow medial concavity and with one pair of denticles; coxopleural sutures complete in entire ventrum, sinuous and diverging anteriorly; chitin-lines absent. Trochanteroprefemur ca 1.3–1.4× as long as wide; with a well-developed and strong pigmented denticle at distal internal margin, ca 1.3–1.6× as long as wide. Intermediate articles distinct, with a tubercle on tibia (arrowed in Fig. 9E, some individuals not visible). Tarsungulum with well-pigmented basal denticle; both external and internal margins uniformly curved, except for moderate mesal basal bulge; ungulum not distinctly flattened. Elongated poison calyx (circle in Fig. 9F), ca 9× as long as wide, lodged inside intermediate forcipular articles.

***Leg-bearing segments*** (Figs 9A, B, 10A–C): Forty-one pairs of legs present. Metatergite 1 slightly wider than subsequent one, with two paramedian sulci visible on tergites of anterior half of body, with pretergite. No paratergites. Walking legs shorter than width of trunk; legs of first pair much smaller than following ones; claws simple, uniformly bent, with 2 accessory spines; anterior spine reaching at most 10% of length of claw; posterior spine equal in length of the anterior spine. Metasternites slightly longer than wide. Sternal sulcus visible on a few anterior sternites, represented by very shallow mid-longitudinal thickening, anteriorly not furcate. No ventral glandular pores on each metasternite.

***Ultimate leg-bearing segment*** (Fig. 11A–D): Pretergite not accompanied by pleurites but incomplete traces of sutures present at both sides. Metatergite subtrapezoidal, ca 1.1–1.3× as wide as long; lateral margins convex and converging posteriorly; posterior margin slightly curved. Coxopleuron ca 1.2–1.7× as long as metasternite; coxal organs of each coxopleuron opening through 5–10 independent pores, placed ventrally. Metasternite trapezoidal, ca 1.3–1.6× as wide as long, anteriorly ca 1.7–2.2× as wide as posteriorly; lateral margins slightly convex and converging backward; setae almost arranged symmetrically, dense on posterior margin. Telopodite ca 9–11× as long as wide, ca 1.9–2.1× as long and ca 1.5–1.6× as wide as penultimate telopodite, with six articles; tarsus 2 ca 3.6–4.2× as long as wide and ca 1.2–1.6× as long as tarsus 1; setae arranged uniformly, < 70 μm long; pretarsus represented by small tubercle.

***Female postpedal segments*** (Fig. 11A, B): Two gonopods basally touching, subtriangular, without traces of articulation, covered with setae. Anal pore present.

Male postpedal segments unknown (male unknown).

###### Distribution.

Honshu, Shikoku and Kyushu.

###### Remarks.

In pairwise comparisons, *N.innuptus* sp. nov. can be distinguished from *N.hoffmani* by the presence of a well-developed denticle on the trochanteroprefemur (width: length = 1:1.3–1.6) and a well-pigmented denticle on the tarsungulum. In addition, *N.innuptus* sp. nov. is also distinguishable from *N.hoffmani* by the presence of a tubercle on the forcipular tibia, but this tubercle is not always visible. No male has been found so far.

##### ﻿ Nannarrup oyamensis

Taxon classificationAnimaliaGeophilomorphaMecistocephalidae

Tsukamoto sp. nov.

1B1A7490-A2C8-5400-8BEF-D6BC3F5DAE32

https://zoobank.org/1543ADD5-1C03-4471-9B6F-D473E4BB0F22

**Figure 12.**
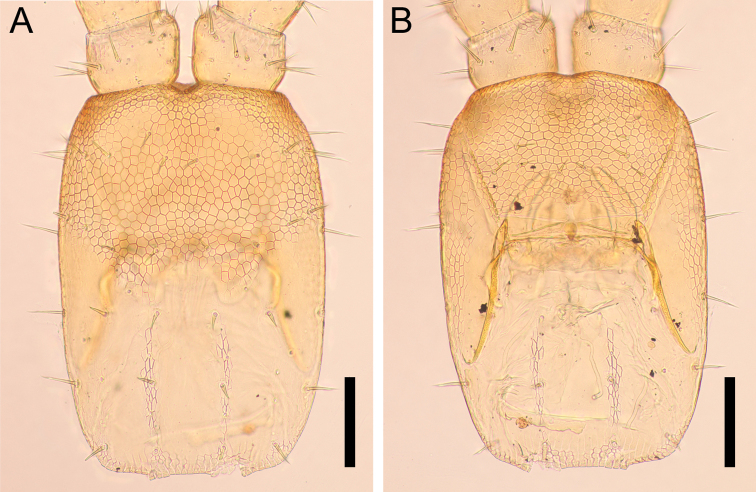
*Nannarrupoyamensis* sp. nov., holotype (TS20210217-04) **A** cephalic plate, dorsal **B** clypeus and clypeal pleurite, ventral. Scale bars: 0.1 mm.

**Figure 13.**
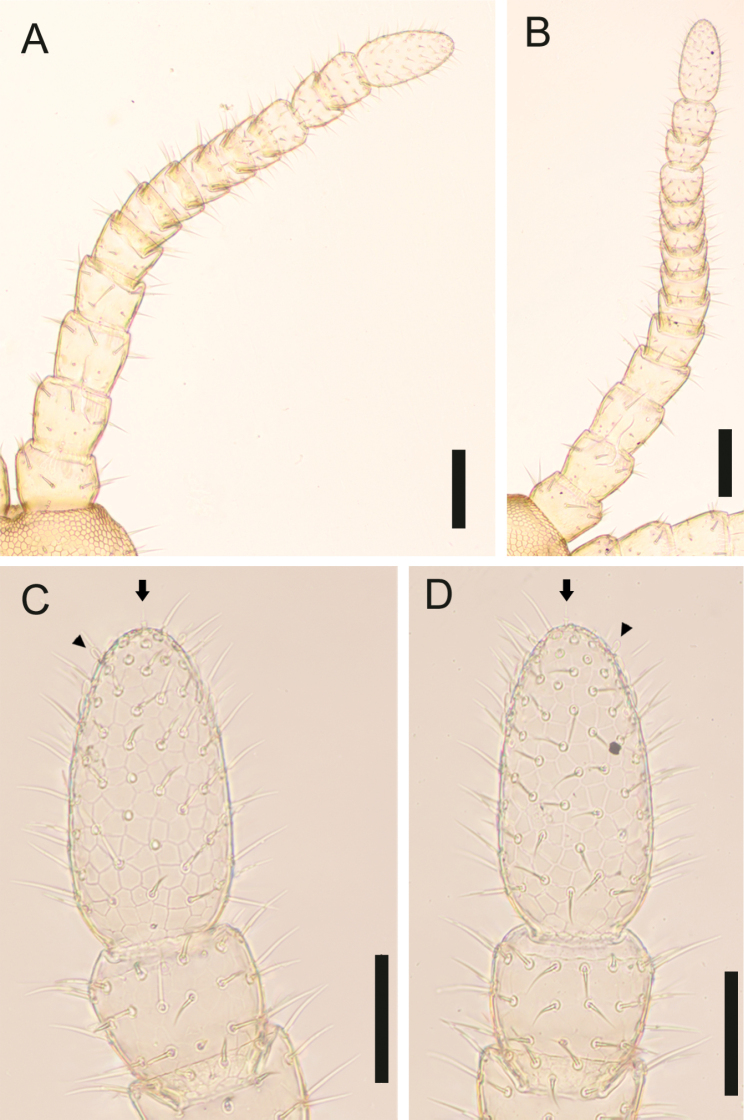
*Nannarrupoyamensis* sp. nov., holotype (TS20210217-04) **A** right antenna, dorsal **B** right antenna, ventral **C** antennal article XIV, dorsal **D** antennal article XIV, ventral. Arrows indicate apical sensillum; arrowheads indicate club-like sensillum. Scale bars: 0.1 mm (**A, B**); 0.05 mm (**C, D**).

**Figure 14.**
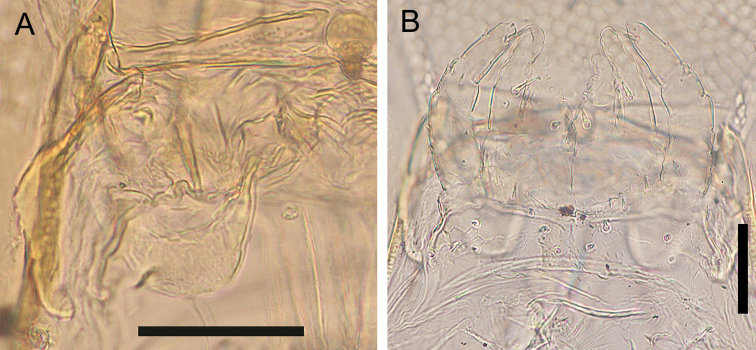
*Nannarrupoyamensis* sp. nov., holotype (TS20210217-04) **A** right mandible, ventral **B** maxillae complex, ventral. Scale bars: 0.05 mm.

**Figure 15.**
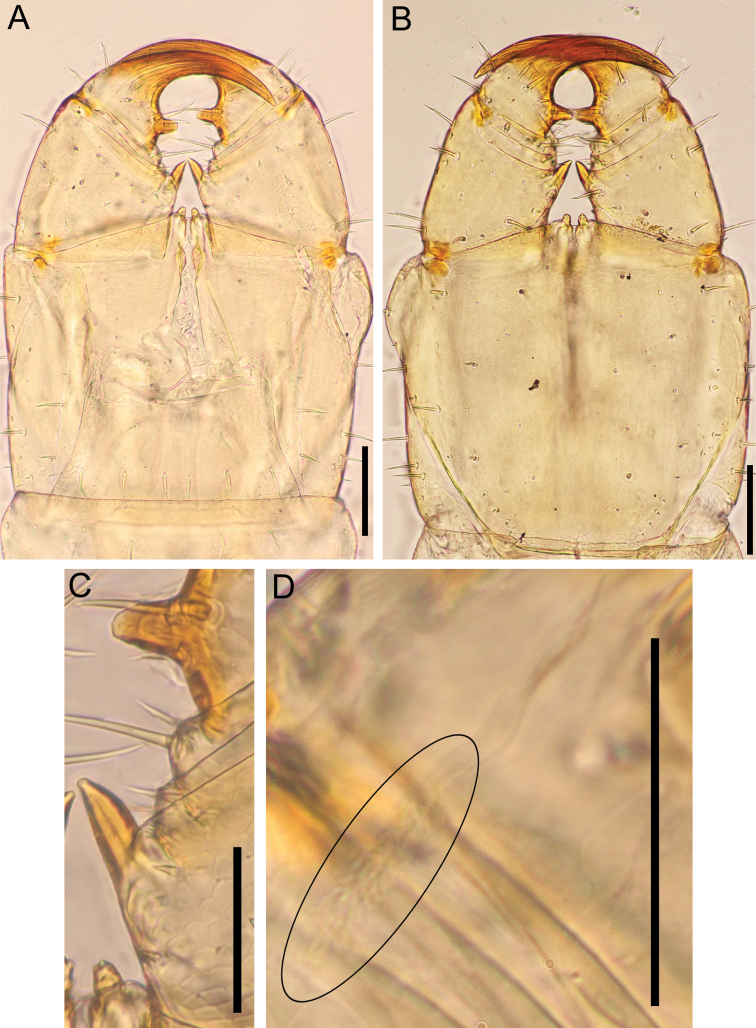
*Nannarrupoyamensis* sp. nov., holotype (TS20210217-04) **A** forcipular segment, dorsal **B** forcipular segment, ventral **C** denticles on right forcipule, dorsal **D** left poison calyx, dorsal. Circle indicates poison calyx. Scale bars: 0.1 mm (**A, B**); 0.05 mm (**C, D**).

**Figure 16.**
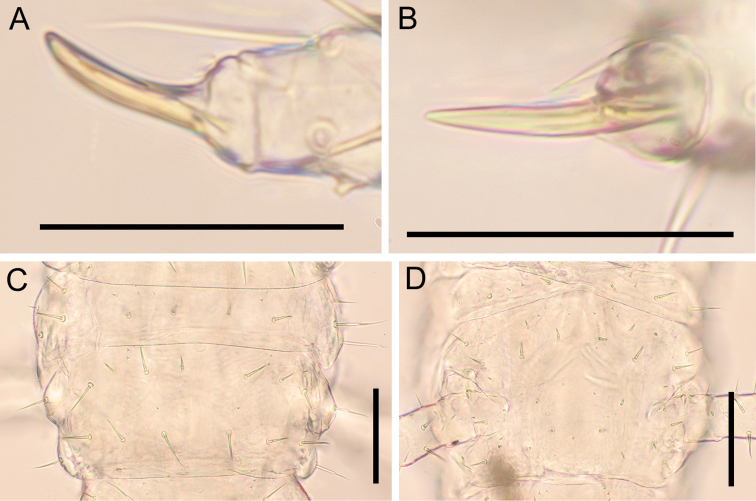
*Nannarrupoyamensis* sp. nov., holotype (TS20210217-04) **A** pretarsus of left leg 40, dorsal **B** pretarsus of left leg 40, ventral **C** tergite of leg-bearing segment 40, dorsal **D** sternite of leg-bearing segment 40, ventral. Scale bars: 0.05 mm (**A, B**); 0.1 mm (**C, D**).

**Figure 17.**
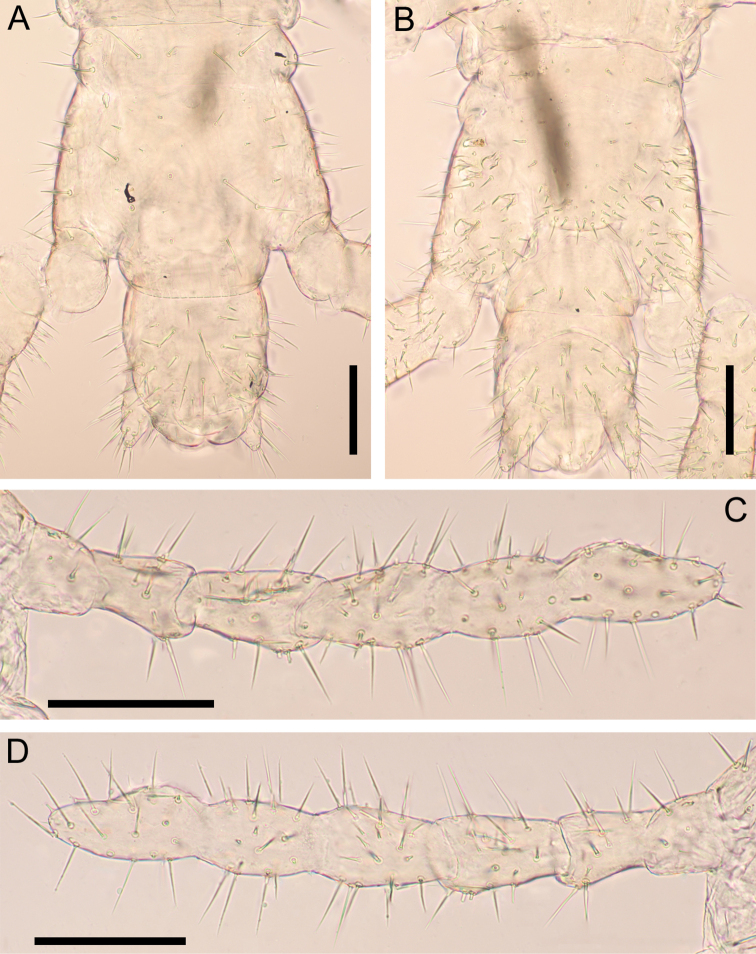
*Nannarrupoyamensis* sp. nov. **A, B** holotype (TS20210217-04) **C, D** paratype (TS20210725-02). **A** ultimate leg-bearing segment and male postpedal segment, dorsal **B** ultimate leg-bearing segment and male postpedal segment, ventral **C** right ultimate leg, dorsal **D** right ultimate leg, ventral. Scale bars: 0.1 mm.

New Japanese name: Amefuri-himejimukade Figs 12 , 13 , 14 , 15 , 16 , 17

###### Type material.

***Holotype*** 1 adult male, Hinata, Isehara-shi, Kanagawa prefecture, Japan (35°26.07'N, 139°14.75'E), 17 February 2021, coll. Sho Tsukamoto (labeled as TS20210217-04), deposited at the Collection of Myriapoda, Department of Zoology, **NSMT**. ***Paratype*** 1 subadult male, Hinata, Isehara-shi, Kanagawa prefecture, Japan (35°26.07'N, 139°14.75'E), 25 July 2021, coll. Sho Tsukamoto (labeled as TS20210725-02), deposited at **MNHAH**.

###### Etymology.

The species name is derived from the name of Japanese mountain, namely Mt. Oyama. The word was further Latinized by adding the Latin masculine adjective suffix -*ensis*, to form oyamensis. The last “a” of Oyama and the first “e” of -ensis are merged into “e.” Examined specimens were collected from Mt. Oyama, an object of the mountain worship.

###### Diagnosis.

Clypeus without smooth or weakly areolate areas along the posterior part of the paraclypeal sutures; forcipular trochantroprefemur with a large denticle (longer than wide); tarsungulum with a well-pigmented denticle; metasternite of ultimate leg-bearing segment wider than long.

###### Description.

***General features***: Body 8.6 mm long (holotype), gradually attenuate posterior, almost uniformly very pale yellow, with head and forcipular segment pale ocher.

***Cephalic capsule*** (Fig. 12A, B): Cephalic plate ca 1.5× as long as wide, lateral margins more distinctly converging anteriorly than posteriorly, posterior margin straight; scutes approximately isometric and up to ca 15 μm wide; transverse suture absent; setae up to ca 50 μm long. Clypeus ca 1.5× as wide as long, with lateral margins complete, almost uniformly areolate, with scutes ca 10 μm wide, a pair of clypeal areas absent; 13 setae in holotype, 1+1 postantennal, 1+1 median, 5+4 prelabral; clypeal ratio ca 1: 7; clypeal plagulae without additional smooth area along posterior part of paraclypeal sutures; 17 pore-like organs on entire part of clypeus. Anterior and distolateral parts of pleurites areolate, without setae. Side-pieces of labrum medially in contact, only incompletely divided into anterior and posterior alae by weak chitinous line, without longitudinal stripes on posterior alae; with slightly visible short fringe on posterior margin of side-pieces; mid-piece as long as wide, converging anteriorly and posteriorly.

***Antenna*** (Fig. 13A–D): Antenna with 14 articles, when stretched, ca 2.3× as long as head length. Intermediate articles slightly longer than wide. Article XIV ca 2.0× as long as wide, ca 1.9× as long as article XIII, and 1.9–2.1× as long as intermediate articles. Setae on articles VIII–XVI denser than articles I–VII. Setae gradually shorter from article VIII to XIV, up to 50 μm long on article I, up to 33 µm long on article VIII and < 18 μm long on article XIV. Article XIV with two types of sensilla, apical sensilla (arrows in Fig. 13C, D) ca 5 μm long, with wide flat ring at mid-length; club-like sensilla (arrowheads in Fig. 13C, D) ca 10 μm long, clustered in the distal parts of the internal and external sides of the article. Three longitudinal rows consisted of ca 9 proprioceptive spine-like sensilla at bases of antennal articles II–V, VII–IX, approximately dorsal, ventro-internal and ventro-external; rows reduced to 1–3 spines on antennal articles I and VI, and 0–1 spine on antennal articles X–XIV. A few pointed sensilla, up to 2.5 μm long, on both dorso-external and ventro-internal position, close to distal margin of articles II, V, IX and XIII.

***Mandible*** (Fig. 14A). At least four pectinate lamellae, with elongated teeth. Each tooth ca 2× as long as wide.

***First maxillae*** (Fig. 14B): Coxosternite medially divided but slightly, without setae, faintly areolate. Coxal projections well developed and hyaline distally, provided with 1+1 setae and 3+4 small sensilla. Telopodite uni-articulated and hyaline distally, with one (two) seta(e). No lobes on either coxosternite or telopodites.

***Second maxillae*** (Fig. 14B): Coxosternite medially undivided, without suture, with 2+3 setae along the anterior margin, with 4+4 setae located behind anterior margin, with anterior margin slightly concave, with metameric pores on posterior part. Telopodites tri-articulate overreaching medial projections and telopodites of first maxillae. Claw of telopodite virtually absent, represented by short spine only.

***Forcipular segment*** (Fig. 15A–D): Tergite trapezoidal, ca 1.9× as wide as long, with lateral margins converging anteriorly, approximately as wide as cephalic plate and ca 0.7× as wide as following tergite, 1+1 setae of similar length arranged in an anterior row, and 3+3 setae of similar length arranged in a posterior row, one pair of longitudinal rows of three tiny setae located between middle and distal setae in posterior row. Mid-longitudinal sulcus of tergite not visible. Exposed part of coxosternite ca 1.3× as wide as long; anterior margin with shallow medial concavity and with one pair of denticles; coxopleural sutures complete in entirely ventrum, sinuous and diverging anteriorly; chitin-lines absent. Basal distance between forcipules ca 0.1× of maximum width of coxosternite. Trochanteroprefemur ca 1.3× as long as wide; with a well-developed and strong pigmented denticle at distal internal margin, ca 1.3× as long as wide. Intermediate articles distinct, tubercle on tibia not visible. Tarsungulum with basal denticle well-pigmented; both external and internal margins uniformly curved, except for moderate mesal basal bulge; ungulum not distinctly flattened. Elongated poison calyx (circle in Fig. 15D), ca 6× as long as wide, lodged inside intermediate forcipular articles.

***Leg-bearing segments*** (Fig. 16A–D): Forty-one pairs of legs present. Metatergite 1 slightly wider than subsequent one, with two paramedian sulci visible on tergites of anterior half of body, without pretergite. No paratergites. Walking legs shorter than width of trunk; legs of first pair much smaller than following ones; claws simple, uniformly bent, with two accessory spines; anterior spine reaching at most 10% of length of claw; posterior spine shorter than anterior spine. Metasternites slightly longer than wide. Sternal sulcus visible on a few anterior sternites, represented by very shallow mid-longitudinal thickening, anterior not furcate. No ventral glandular pores on each metasternite.

***Ultimate leg-bearing segment*** (Fig. 17A–D): Pretergite not accompanied by pleurites but incomplete traces of sutures present at both sides. Metatergite subtrapezoidal, almost as wide as long, lateral margins convex and converging posteriorly; posterior margin slightly curved. Coxopleuron ca 1.2× as long as metasternite; coxal organs of each coxopleuron opening through five or six independent pores, placed ventrally. Metasternite subtriangular, ca 1.6 as wide as long, anteriorly ca 3.5× as wide as posteriorly; lateral margins slightly convex and converging backward; setae almost arranged symmetrically, dense on posterior margin. Telopodite ca 11× as long as wide, ca 1.9× as long and ca 1.3× as wide as penultimate telopodite, with 6 articles; tarsus 2 ca 2.7× as long as wide and ca 1.5× as long as tarsus 1; setae arranged uniformly, < 50 μm long. Pretarsus represented by spines, up to 5 µm.

***Male postpedal segments*** (Fig. 17A, B): Two gonopods, very widely separated from one another, conical in outline, uni-articulated without any sutures, covered with setae. Anal pore present.

Female postpedal segments unknown (female unknown).

###### Distribution.

Only known from Mt. Oyama, located in Isehara-shi, Kanagawa prefecture.

###### Remarks.

*Nannarrupoyamensis* sp. nov. is distinguishable from the two congeners by the absence of smooth or weakly areolate areas along the posterior part of the paraclypeal sutures. Specifically, *N.oyamensis* sp. nov. can be clearly distinguished from *N.hoffmani* by the presence of a well-developed denticle on the trochanteroprefemur (width: length = 1:1.3) and the absence of smooth or weakly areolate areas along the posterior part of the paraclypeal sutures. Furthermore, *N.oyamensis* sp. nov. can be distinguished from *N.innuptus* sp. nov. by the absence of a pair of smooth or weakly areolate areas along the posterior part of the paraclypeal sutures (see Table 3 for a comparison of characteristics).

## ﻿Discussion

### ﻿Species recognition based on morphological analysis and DNA barcoding

All three morphospecies (including *N.hoffmani*) were determined to be similar to a certain extent. Nevertheless, each morphospecies was distinguished from the other two morphospecies on the basis of the following characteristics: presence or absence of a pair of smooth or weakly areolate areas along the posterior part of the paraclypeal sutures, the width-to-length ratio of the denticle of trochanteroprefemur, the pigmentation of the denticle on the tarsungulum. Table 3 shows the comparison among the three morphospecies regarding the key characters.

The maximum internal genetic divergence in the *COI*, *16S*, and *28S* sequences within each morphospecies was considerably smaller than the minimum divergence in the *COI*, *16S*, and *28S* sequences between each pair of the morphospecies, that is, the DNA barcoding gap was evident.

The presence of a DNA barcoding gap (especially in the nuclear *28S*) suggests that *N.* sp. 2 forms an independent gene pool that is reproductively isolated from *N.* sp. 1. Together, the morphological and molecular evidence suggests that the three morphospecies are distinct species and that *N.* sp. 1 and *N.* sp. 2 are two novel species; they are described under the section “Taxonomic account” as *Nannarrupinnuptus* sp. nov. and *Nannarrupoyamensis* sp. nov., respectively.

### ﻿Distribution of the genus *Nannarrup*

All examined *Nannarrup* specimens were collected from Honshu, Shikoku, and Kyushu, but the collectors (ST, KE, and collaborators) did not find *Nannarrup* in the Ryukyu Archipelago despite intensive and repetitive field surveys. Furthermore, Uliana et al. (2007) have also reported that there are no records of *Nannarrup* from the Ryukyus and Taiwan. The distribution range of *Nannarrup* is likely confined to the temperate zones of East Asia, namely Honshu, Shikoku, Kyushu, and surrounding islands. However, future field surveys in Hokkaido, and continental East Asia may lead to additional reports of *Nannarrup*.

Foddai et al. (2003) stated that *N.hoffmani* is not native to New York, the type locality of this species, but has been definitely introduced from western America or East Asia. In the present study, *N.hoffmani* was not collected in Japan. However, the existence of two congeners in Japan suggests that *Nannarrup* is native to East Asia. Additional field surveys to investigate the original distribution of *N.hoffmani* should be conducted mainly in East Asia.

According to the results of DNA barcoding based on *COI*, *16S*, and *28S* sequences, *N.innuptus* sp. nov. does not show a remarkable genetic structure that is consistent with the geography of Japan and physical distances among the collection sites in the present study. Nevertheless, considering this intraspecific genetic diversity, it is reasonable to assume that *N.innuptus* sp. nov. is not a species recently introduced by human activities but a native species in Japan. The reason for such a low genetic diversity and no geographic structuring of diversity in mitochondrial genes remains unclear at present, but we propose the following two hypotheses to explain this finding: 1) the rate of evolution of mitochondrial genes is considerably lower than that in other centipedes, and 2) the establishment of *N.innuptus* sp. nov. as an independent species (either within the Japanese archipelago or following migration from the Asian continent) is geologically relatively recent. This question may be answered by examining sufficient specimens of other congeners (e.g., *N.oyamensis* sp. nov.) from East Asia including Japan and estimating the species divergence time by including other congeners and even genera belonging to Mecistocephalidae in Asia.

### ﻿Likelihood of parthenogenesis in *Nannarrupinnuptus* sp. nov.

Remarkably, all 71 adult and subadult specimens of *N.innuptus* sp. nov. examined in this study were females without exception. Furthermore, because different collectors (four) did the specimen collections, in different seasons (March–November), and in different habitats (forest, urban green space, and riverbed), the presence of any sampling bias that may have distorted the sex ratio is unlikely. Although there is no direct evidence, the abovementioned indirect evidence shows the possibility of parthenogenesis of *N.innuptus* sp. nov.

To date, a large part of the northern and eastern European population of *Geophilusproximus* C.L. Koch, 1847, the European population of *Tygarrupjavanicus* Attems, 1929, and *Schendyladentata* (Brölemann & Ribaut, 1911) have been discussed about their parthenogenesis (Sograff 1882; Barber and Jones 1999; Minelli 2011; Tuf et al. 2018). *Nannarrupinnuptus* sp. nov. may be the second parthenogenetic species in the family Mecistocephalidae. In contrast, the Japanese congener, *N.oyamensis* sp. nov., exhibits sexual reproduction because two examined specimens were males, i.e., males of *N.oyamensis* sp. nov. should be common. In addition, the type series of *N.hoffmani* involved one juvenile male specimen (Foddai et al. 2003).

## Supplementary Material

XML Treatment for Nannarrup

XML Treatment for Nannarrup innuptus

XML Treatment for Nannarrup oyamensis
